# Tracheal adenoid cystic carcinoma: a case report

**DOI:** 10.3389/fonc.2025.1671330

**Published:** 2025-10-09

**Authors:** Sawsen Dhambri, Wejdan Mohamed Trabelsi, Rim Braham, Mohamed Dhaha, Khadija Ben Zid, Chiraz Nasr, Skander Kedous

**Affiliations:** Otorhinolaryngology – Head and Neck Surgery, Institut Salah Azaiez, Tunis, Tunisia

**Keywords:** tracheal neoplasms, radiotherapy, surgery, adenoid cystic carcinoma, perineural invasion, adjuvant therapy, airway reconstruction

## Abstract

**Background:**

Primary malignant tumors of the trachea are extremely rare, representing only 0.2% of all respiratory tract tumors. Among them, adenoid cystic carcinoma (ACC) is the second most frequent histological type. Due to its slow growth and misleading symptoms, diagnosis is often delayed.

**Case:**

We report the case of a 42-year-old male, chronic smoker, who presented with progressive dyspnea and mild hemoptysis. Chest CT revealed a 32-mm expansive lesion of the cervical trachea causing 80% stenosis. Bronchoscopy confirmed an intratracheal mass, and biopsy established the diagnosis of tracheal adenoid cystic carcinoma (TACC).

**Management:**

The patient underwent crico-tracheal resection with total thyroidectomy and central neck dissection. Histopathology confirmed TACC with perineural and angiolymphatic invasion, as well as thyroid gland infiltration, but clear surgical margins. Adjuvant radiotherapy was delivered using volumetric modulated arc therapy to a total dose of 60 Gy.

**Outcome:**

The postoperative course was uneventful, and the patient recovered well. At one-year follow-up, both CT and bronchoscopy confirmed an unobstructed trachea with no evidence of local recurrence or distant metastasis.

**Conclusion:**

TACC is an uncommon and challenging malignancy requiring multidisciplinary management. Radical surgery with adjuvant radiotherapy remains the optimal strategy to achieve local control, particularly in cases with perineural invasion. Early diagnosis and complete resection are key to improving long-term prognosis.

## 1 Introduction

Primary malignant tumors of the trachea are extremely rare, accounting for only 0.2% of all respiratory tract neoplasms (1, 2). Among these, adenoid cystic carcinoma (ACC), formerly termed cylindroma, represents the second most common histological subtype, with an estimated incidence of 0.04 to 0.2% (1, 2). This tumor arises from the intraductal submucosal seromucinous glands of the trachea and bronchi (3) and typically presents in relatively young adults, without gender predilection and without association with tobacco use (4).

Despite its indolent growth, tracheal ACC is diagnostically challenging. Clinical manifestations such as cough, wheezing, dyspnea, and mild hemoptysis are nonspecific and often mimic more common respiratory disorders such as asthma, chronic bronchitis, or recurrent pneumonia (5). This symptomatic overlap frequently leads to diagnostic delays, with several studies reporting an average interval of up to one year before accurate recognition. Conventional chest radiography is frequently unremarkable, while computed tomography and bronchoscopy remain essential for detecting tracheal stenosis and obtaining tissue samples (6). Cytological diagnosis may also be difficult since the tumor is often covered by intact mucosa, requiring histological confirmation of its characteristic cribriform architecture (7).

Therapeutic management of tracheal ACC is also complex. Radical surgical resection with airway reconstruction is considered the treatment of choice (8), yet the infiltrative nature of this tumor, with its tendency for perineural and angiolymphatic spread, often results in positive surgical margins (9). Postoperative radiotherapy is therefore widely used to improve local control, especially in cases with incomplete resection or histological evidence of high-risk features.

The present case is noteworthy because it highlights two uncommon patterns of extension: invasion of the thyroid gland and involvement of the recurrent laryngeal nerve. Such presentations are rarely described in the literature, and they emphasize the aggressive potential of this otherwise slow-growing malignancy. By reporting this case, we aim not only to illustrate the diagnostic challenges and therapeutic strategies involved in tracheal ACC, but also to contribute to the limited body of knowledge regarding unusual patterns of invasion and their clinical implications.

## 2 Case presentation

42-year-old man, smoker of 20 packs/year, consulted for progressive dyspnea and mild hemoptysis that had appeared five weeks previously. There was no fever or bronchopulmonary history. Tomodensitometry (CT) of the chest revealed an expansive lesion of the cervical trachea below the cricoid cartilage, extending over 32 mm, and causing an 80% stenosis of the tracheal lumen. The mass appears to extend beyond the posterior wall, without evidence of cricoid cartilage lysis. Rigid bronchoscopy visualized the tumor and biopsy confirmed the diagnosis of cystic adenoid carcinoma. The case was reviewed in a multidisciplinary medical meeting and surgery was decided.

**Figure 1:**
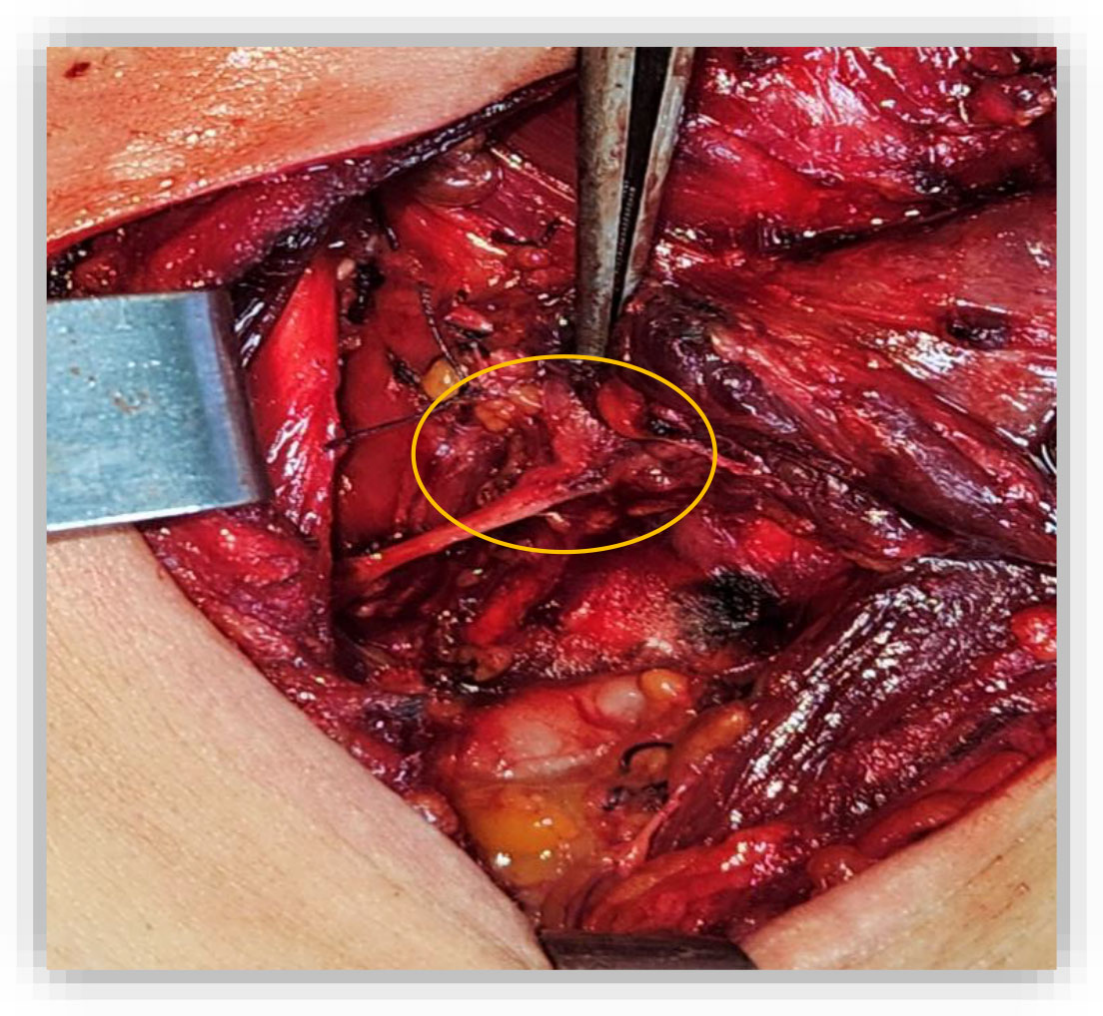
Intraoperative view showing tumor invasion of the right recurrent laryngeal nerve. The whitish friable mass (orange cercle), can be seen extending along the right lateral tracheal wall, necessitating sacrifice of the nerve to achieve complete resection.

**Figure 2:**
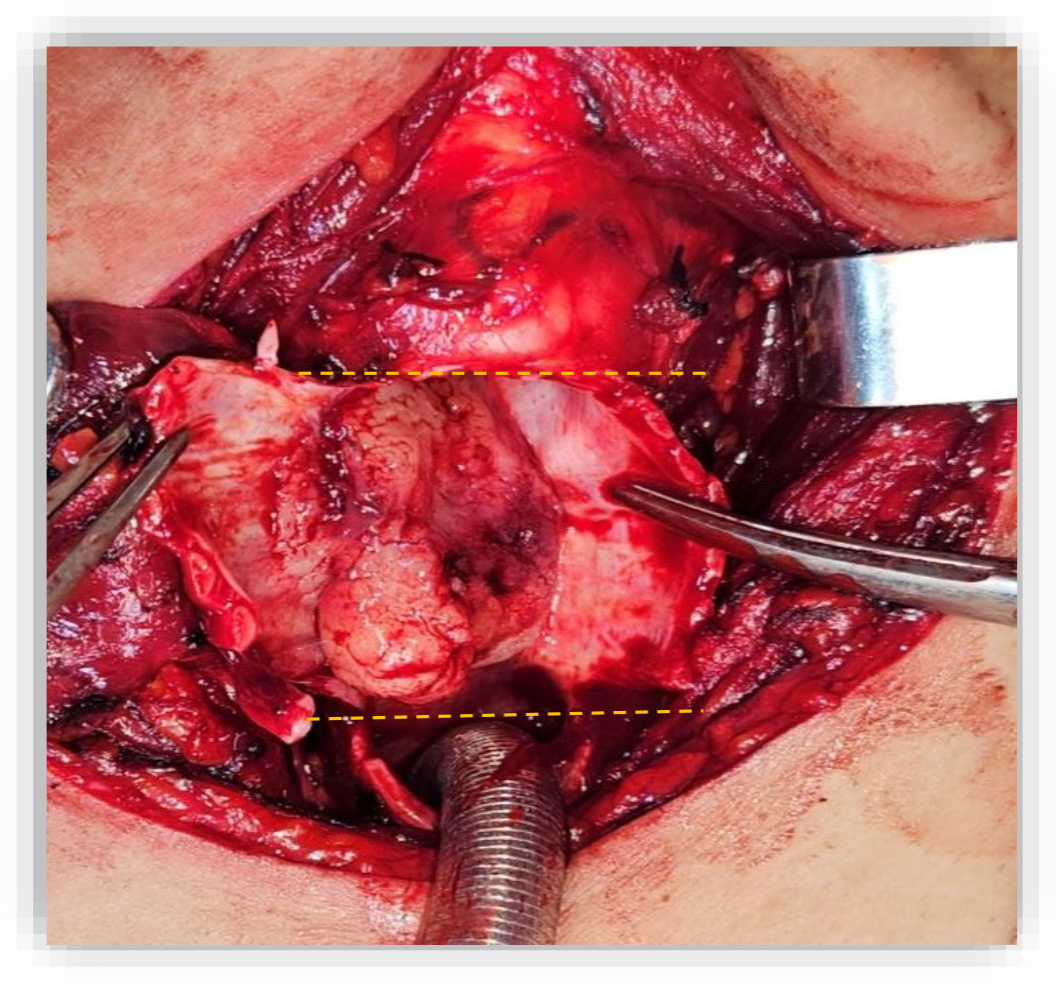
Resected tracheal specimen illustrating the superior and inferior margins (Dashed lines). The tumor involved the cricoid cartilage and extended from the fourth tracheal ring up to the crico-thyroid membrane.

On May 30, 2023, A partial crico-tracheal resection with total thyroidectomy and central neck dissection was performed under general anesthesia. A kocher neck incision was made and the thyroid lobes were mobilized. The recurrent laryngeal nerves were identified bilaterally. On exposure of the trachea, a crumbly, whitish mass was encountered in the right lateral wall of the trachea, which extended from the 4th ring to the crico-thyroid membrane, with invasion of the recurrent laryngeal nerve on the right side (**Figure 1**). Intraoperatively, the intratracheal mucosa in this region appeared markedly friable, with a fragile consistency and a tendency to bleed upon minimal manipulation. After opening the specimen, the mucosal extension into the airway lumen was found to be more extensive than initially appreciated. Resection was therefore carried out down to the level of the sixth tracheal ring (**Figure 2**). A mucosal flap from the seventh tracheal ring was obtained and submitted for frozen section analysis, which confirmed the absence of tumor infiltration. Additionally, intraoperative assessment revealed tumoral extension involving the mucosa of the inferior border of the cricoid cartilage. Total laryngectomy wasn’t performed due to clean margins but the right recurrent laryngeal nerve, which had been invaded by the tumor, was sacrificed and a bilateral central neck dissection was carried out. In view of the intraoperative finding of tumor invasion into the right thyroid lobe and the recurrent laryngeal nerve, a partial crico-tracheal resection with total thyroidectomy and central neck dissection was undertaken. This approach was considered optimal to achieve oncologically safe margins while maintaining airway stability and functional reconstruction. Primary single-stage tracheal reconstruction was achieved without the requirement for tracheostomy. A tension-free anastomosis was obtained through careful mediastinal tracheal mobilization combined with partial laryngeal release. The decision to include thyroidectomy was guided by direct tumor invasion, while total laryngectomy was avoided since frozen sections confirmed clear proximal and distal margins. The postoperative course was uneventful. The cervical Redon drainage system was removed on post-operative day 3. The patient was discharged after 12 days with corticotherapy for five days. The patient recovered well after the operation.

Definitive histopathology revealed the characteristic cribriform and tubular growth patterns of adenoid cystic carcinoma, composed of basaloid epithelial cells arranged around pseudocystic spaces filled with hyaline material. Perineural and angiolymphatic invasion were clearly identified, consistent with the locally aggressive nature of the lesion. Immunohistochemistry (IHC) confirmed that the tumor expressed cytokeratins (CK7), smooth muscle actin (SMA) and p63, while lacking expression of TTF-1 and thyroglobulin. The final diagnosis was adenoid cystic carcinoma with clear microscopic margins and infiltration of the cricoid cartilage, the right thyroid gland, and perineural as well as angiolymphatic structures. Three reactive lymph nodes were also identified.

**Figure 3:**
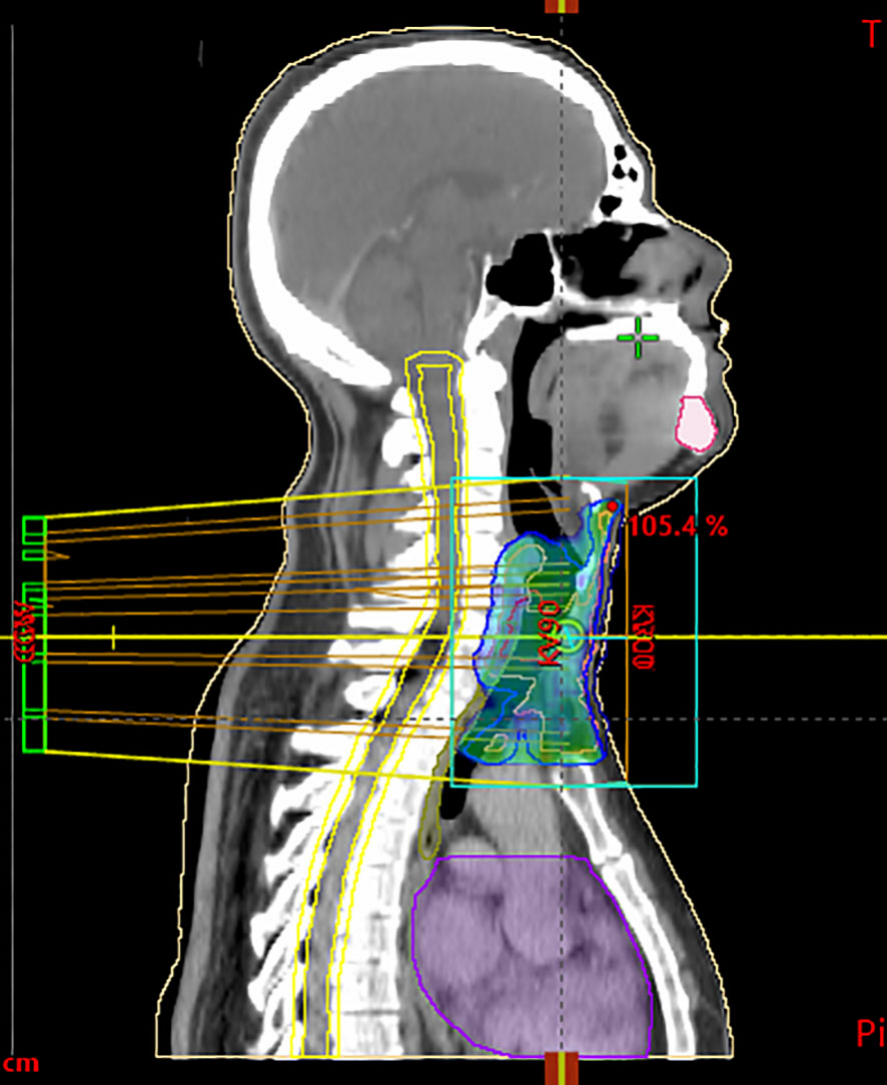
Sagittal CT scan used for radiotherapy planning, showing target volumes and isodose distribution. The blue/green areas represent the clinical target volume (CTV) and planning target volume (PTV), including the primary tracheal tumor and safety margins. Yellow lines delineate isodose curves, illustrating the percentage of the prescribed dose (95%, 100%, 105%) delivered to the tumor region. The violet contour corresponds to an organ at risk (OAR), delineated to monitor dose exposure.

According to the pathological exam, adjuvant radiotherapy was decided at a multidisciplinary team meeting. The clinical target volume was delineated as the gross tumor volume with a 1-cm margin, including the mediastinal structures. A dose of 60 Gy for planning treatment volume in fractions of 2 Gy was administered based on 6-MV X-ray with volumetric modulated arc therapy. Maximum spinal cord dose was 29.5 Gy (**Figure 3**). Volume of the esophagus receiving 60 Gy was 2.5% and a mean dose of 38.7Gy. Radiotherapy was completed in August 2023, and since then, the patient remains in observation.

The postoperative course was uneventful, and the patient recovered well. Functional outcomes were satisfactory: airway stability was maintained, swallowing was preserved, and the patient reported only mild hoarseness due to sacrifice of the right recurrent laryngeal nerve, without aspiration or significant impact on daily communication. One year after surgery, CT and bronchoscopy showed that the trachea was unobstructed and there was no recurrence of the tumor.

## 3 Discussion

Tracheal neoplasms occur at a rate of 0.1 cases per 100.000 individuals annually, representing only 0.2% of all respiratory tracts tumors (10). Among the primary malignant tracheal tumors, ACC is an infrequent tumor that accounts for 0.1% of respiratory cancers (11).This pathology develops most commonly in the upper posterior trachea. The main symptoms are correlated with tracheal obstruction such as coughing wheezing and dyspnea, that are usually misdiagnosed considering the young age (12). ACC have a favorable prognosis with a five year overall survival of 70% (13).

Our surgical approach illustrates the principle that complete resection provides the best long-term outcome in tracheal ACC. Invasion of the thyroid gland and the recurrent laryngeal nerve, though rare, has been reported in previous series, and mandates extended resection to ensure oncologic completeness while maintaining functional airway reconstruction (6, 14).

The invasion and metastasis of Tracheal adenoid cystic carcinoma are the main reasons for a poor prognosis. It mostly spreads through local infiltration, submucosal or peripheral nerve infiltration, and hematogenous metastasis. More than 50% of TACC cases have hematogenous metastasis. The most common site is the lung, with metastasis also found in the brain, bone, liver, kidney, skin, abdomen, and heart. The invasion of TACC into the thyroid gland is very rare and is typically misdiagnosed. The TACC mainly invades the thyroid by local infiltration with few cases of hematogenous metastasis. In our case, it involved the right side of thyroid gland and the right recurrent laryngeal nerve, so complete resection of all thyroid tissue and nerve was performed.

For TACC, radical surgical resection is the most effective treatment option. The 5-year survival rate of patients undergoing surgery is 85%, while that of patients who do not undergo surgery is 63.7% (15). Since TACC often infiltrates to the periphery and especially tends to infiltrate the peripheral vascular and nerve bundles, the surgical boundary is difficult to determine, and positive postoperative margins are common. Positive margins have been reported to occur in 15% of cases that are confined to the tracheal wall and 85% of cases that extend into the tracheal wall (6). Positive margins are the most important risk factors for recurrence and metastasis (14). The pathological examination after surgery in the present case also showed that the nerve was involved. In order to reduce the risk of recurrence, we performed an extended resection. The surgical approach for such tumors depends on the surgeon’s experience, the size and location of the tumor, and the degree of local invasion (16). The extent of surgical resection is limited by tracheal length, and it is generally accepted that the resected length cannot exceed 6 cm or 50% of the total length. Xie et al. (17) reported a case of TACC with a tumor length of 6.5 cm. They removed approximately 7.5 cm of the tracheal membranous wall (about 64.1% of the total length of the trachea). To reduce the anastomotic tension, they used a pedicled pectoralis major myocutaneous flap to repair the gap in the tracheal wall. Tracheal adenoid cystic carcinoma is usually operated on by direct surgical resection of the trachea and airway reconstruction, while Jiao et al. (16) introduced a new technique of thoracoscopic tracheal resection with end-to-end anastomosis, which is less invasive. However, it is limited to lesions of the lower trachea or bronchus, and difficult airway reconstruction cannot be completed, so it is not suitable for the patient described in this report. The main surgical complications include tracheoesophageal fistula, hypopharyngeal fistula, poor anastomotic healing, and vocal cord paralysis, resulting in difficulty in expectoration and pulmonary infection often requiring tracheotomy (18);

The role of radiotherapy is still unclear. It is usually offered as an adjuvant treatment for patients with incomplete resection or inoperable with local control rates ranging from 20 to 70% (19, 20). There are no randomized prospective clinical trials applying this approach. In a 30-year retrospective study, Calzada et al. (21) observed a higher disease-free survival rate among patients who underwent surgery with adjuvant radiation regardless of resection limits. The recommended dose for adjuvant radiotherapy is 60 Gy in fractions of 2 Gy (22, 23). In our case, radiotherapy was indicated for perineural and angiolymphatic invasion in the definitive histopathology. Beyond the immediate therapeutic outcome, long-term challenges remain a major concern in tracheal ACC (19). Local recurrences may arise more than a decade after apparently complete resection, underscoring the need for lifelong surveillance with imaging and endoscopic follow-up. In addition, late complications of adjuvant radiotherapy must be anticipated, including airway fibrosis and radiation-induced stenosis, which can compromise long-term functional outcomes and quality of life (24).

## 4 Conclusion

Tracheal adenoid cystic carcinoma is a rare malignancy that often presents with nonspecific symptoms, leading to diagnostic delays. Surgery remains the mainstay of treatment, though it is frequently challenging due to the tumor’s infiltrative nature and involvement of adjacent structures. Adjuvant radiotherapy is essential in cases with perineural or angiolymphatic invasion, as it improves locoregional control. Despite favorable short-term outcomes, long-term follow-up is mandatory since recurrence may occur even 10 to 15 years after initial treatment. Although our patient remains disease-free at one-year, long-term vigilance is essential. Tracheal ACC is prone to late local recurrence, even after initial treatment, and adjuvant radiotherapy may induce delayed toxicities such as fibrosis and tracheal stenosis. These risks highlight the importance of close, lifelong follow-up to promptly detect recurrence and manage late sequelae. Future studies focusing on molecular characterization and new therapeutic strategies are needed to better guide the management of this uncommon disease.

## Data Availability

The data presented in the study are deposited in the Zenodo repository, accession number :10.5281/zenodo.17236502.
